# Flux Analysis Uncovers Key Role of Functional Redundancy in Formaldehyde Metabolism

**DOI:** 10.1371/journal.pbio.0030016

**Published:** 2005-01-04

**Authors:** Christopher J Marx, Stephen J Van Dien, Mary E Lidstrom

**Affiliations:** **1**Department of Microbiology, University of WashingtonSeattle, WashingtonUnited States of America; **2**United MetabolicsSeattle, WashingtonUnited States of America; **3**Department of Chemical Engineering, University of WashingtonSeattle, WashingtonUnited States of America; University of MichiganUnited States of America

## Abstract

Genome-scale analysis of predicted metabolic pathways has revealed the common occurrence of apparent redundancy for specific functional units, or metabolic modules. In many cases, mutation analysis does not resolve function, and instead, direct experimental analysis of metabolic flux under changing conditions is necessary. In order to use genome sequences to build models of cellular function, it is important to define function for such apparently redundant systems. Here we describe direct flux measurements to determine the role of redundancy in three modules involved in formaldehyde assimilation and dissimilation in a bacterium growing on methanol. A combination of deuterium and ^14^C labeling was used to measure the flux through each of the branches of metabolism for growth on methanol during transitions into and out of methylotrophy. The cells were found to differentially partition formaldehyde among the three modules depending on the flux of methanol into the cell. A dynamic mathematical model demonstrated that the kinetic constants of the enzymes involved are sufficient to account for this phenomenon. We demonstrate the role of redundancy in formaldehyde metabolism and have uncovered a new paradigm for coping with toxic, high-flux metabolic intermediates: a dynamic, interconnected metabolic loop.

## Abstract 2

By feeding bacteria methanol labeled with deuterium, the relative contributions of two distinct pathways of formaldehyde metabolism have been demonstrated

## Introduction

The availability of large numbers of genome sequences has facilitated metabolic reconstruction based on predicted gene function, in essence, a prediction of the metabolic blueprint of a cell. Such metabolic reconstructions [1,2,3] can be grouped in functional segments, or metabolic modules [4,5], and the compilation of metabolic modules can be used to predict interactions between the different elements of the metabolic network in a cell. However, a major difficulty with this approach is the common occurrence of apparently redundant functional modules. It is often not possible to assign roles to these metabolic segments, which have been referred to as the “gray areas of the genome” [6]. Expression profiling, either of transcripts or proteins, holds the promise to gain more insight into the function of redundant metabolic modules, but the presence of a transcript or protein does not necessarily correlate with module function, due to posttranslational effects on metabolic flux. In order to determine the true function of such metabolic modules, it is necessary to measure the flux of metabolites through each functional module during relevant physiological changes.

One system that has proved amenable to a modular approach to metabolism is the ability to grow on one-carbon (C_1_) compounds, or methylotrophy [7]. The availability of a gapped genome sequence for a model methylotrophic bacterium, Methylobacterium extorquens AM1, has accelerated the definition of methylotrophy modules, and a reasonably complete metabolic reconstruction is available for this bacterium [7]. However, these analyses coupled to genetic and physiological studies [8,9,10,11,12,13] have raised a series of fundamental questions that can only be answered through direct flux measurements.

**Figure 1:**
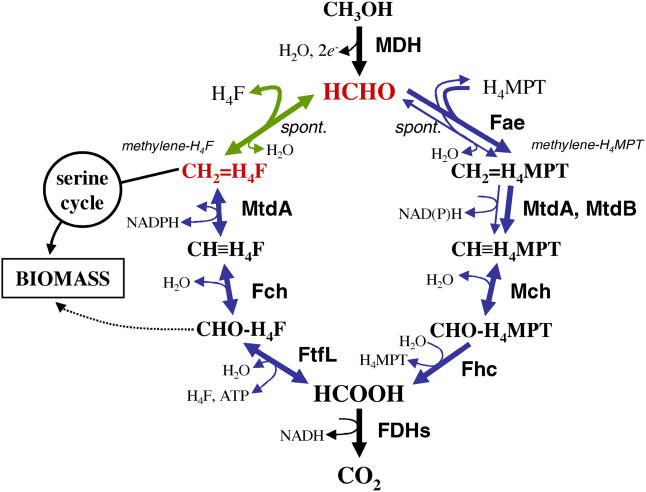
Formaldehyde Metabolism of M. extorquens AM1 Three modules work to provide two cellular outputs: formaldehyde assimilation and dissimilation. The direct condensation of formaldehyde with H_4_F is shown in green. A second proposed route for generating methylene-tetrahydrofolate (methylene-H_4_F), the consecutive action of the H_4_MPT and H_4_F modules is shown in blue. Fae, formaldehyde activating enzyme; Fch, methenyl H_4_F cyclohydrolase; FDH, formate dehydrogenase; Fhc, formyltransferase/hydrolase complex; FtfL, formyl H_4_F ligase; H_4_MPT, tetrahydromethanopterin; Mch, methenyl H_4_MPT cyclohydrolase; MDH, methanol dehydrogenase; MtdA, methylene H_4_F/H_4_MPT dehydrogenase; MtdB, methylene H_4_MPT dehydrogenase. Spontaneous and reversible reactions are indicated.

As in other such aerobic methylotrophic bacteria, M. extorquens AM1 oxidizes C_1_ substrates to formaldehyde and is essentially growing on formaldehyde for both carbon and energy metabolism [14] (Figure 1). It is not yet understood how the toxic central metabolite formaldehyde is efficiently and dynamically partitioned between assimilatory and dissimilatory metabolism, without toxic buildup. Therefore, this system represents both a key problem of methylotrophy and a paradigm for how toxic metabolites are managed in high-flux conditions. Genomic predictions and mutant analyses have identified three functional modules that direct formaldehyde into two outputs: assimilatory or dissimilatory metabolism (Figure 1). The first module consists of the apparently nonenzymatic condensation reaction between formaldehyde and tetrahydrofolate (H_4_F) [9,15] to generate methylene-H_4_F directly, which is the C_1_ donor for assimilation via the serine cycle. The second module is initiated by an enzyme-catalyzed reaction [9] of formaldehyde with a folate compound found in methanogenic Archaea, tetrahydromethanopterin (H_4_MPT). The resulting methylene-H_4_MPT is subsequently oxidized through a series of reactions to formate [8,16,17], which can ultimately be dissimilated to CO_2_ via the activity of multiple formate dehydrogenases [18]. Finally, a third module involves interconversion of methylene-H_4_F and formate via a familiar set of H_4_F-dependent reactions found in most organisms [11,19,20]. Mutant analysis has shown that both the H_4_MPT and H_4_F modules are required for growth on C_1_ compounds [8,9,10,11,12,13,19].

Two distinct models exist to explain the necessity of both the H_4_MPT and H_4_F modules in methylotrophy, predicting opposite directions for the net flux through the H_4_F module. It was suggested over 20 y ago that the H_4_F module functions in formaldehyde oxidation [21]. This predicts that the H_4_MPT and H_4_F modules are parallel, redundant formaldehyde oxidation systems. Recent genetic and biochemical evidence [11,12,13], however, suggest that the H_4_F module is not functionally redundant to the H_4_MPT module for formaldehyde oxidation. An alternative hypothesis suggests that the H_4_F module functions in the reductive direction, generating methylene-H_4_F from formate [11,16,17]. This model suggests a single dissimilatory module (H_4_MPT module) and two, redundant assimilatory modules: the H_4_F module and the direct condensation of methylene-H_4_F from formaldehyde (Figure 1, green arrows). This model predicts two routes for generating the key assimilatory intermediate methylene H_4_F from formaldehyde: one we will term “direct,” involving the direct condensation step, and one we will term “long,” involving the consecutive action of the H_4_MPT and H_4_F modules. Although the direct route (Figure 1, green arrows) requires flux through a nonenzymatic reaction, assimilation via the proposed long route (Figure 1, blue arrows) involving the action of the H_4_MPT and H_4_F modules is energetically costly due to a net expenditure of one ATP per C_1_ unit. If this hypothesis is correct, the H_4_MPT module would play a role in both dissimilatory and assimilatory metabolism, in much the same way that the tricarboxylic acid cycle plays a dual role in growth on multicarbon compounds.

Clearly, this is an example in which metabolic reconstruction is not sufficient to predict the roles of the central metabolic modules involved in carbon partitioning. In addition, it provides a test case for how cells cope with a high-flux toxic metabolic intermediate. In order to address this problem, we have used a combination of stable isotope- and radioisotope-labeling approaches, which has allowed the complete determination of flux through every branch of methylotrophy. The results provide a dynamic picture of the response of M. extorquens AM1 during transitions in and out of methylotrophy. Furthermore, a kinetic model of the key formaldehyde utilization systems was developed that successfully predicted key system dynamics. Our data resolve the specific roles for three interconnected metabolic modules that have two cellular outputs, assimilation and dissimilation. Furthermore, we have revealed a new paradigm for handling high-flux toxic intermediates: a dynamic metabolic loop that demonstrates graded response to changing metabolic needs.

## Results

### Detection of Serine-Derived Mass Fragments Using Gas Chromatography–Mass Spectrometry

**Figure 2:**
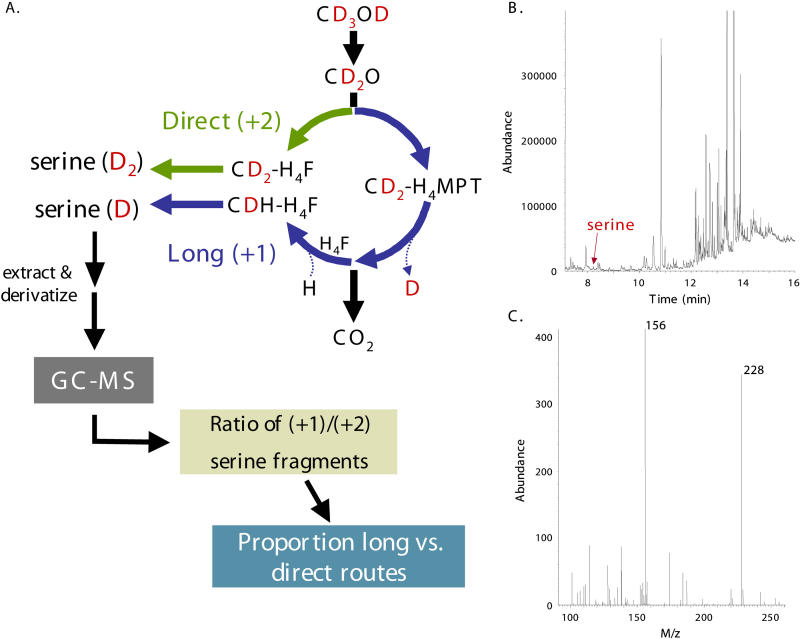
GC–MS Method to Assay Ratio of Long Versus Direct Routes (A) Simplified model of formaldehyde metabolism highlighting the deuterium (in red) label-tracing strategy. Oxidation of deuterated methanol (CD_3_OD) leads to the production of formaldehyde with two deuteriums (CD_2_O). Direct condensation with H_4_F (green arrows) and conversion to serine via the serine cycle (Figure 1) generates serine with two deuteriums. Alternatively, methylene-H_4_F may be produced through the long route (blue arrows; Figure 1), generating serine containing only one of the original deuteriums. Extraction and derivatizion of small molecules for analysis by GC–MS provides the ratio of (+1)/(+2) serine isotopomers, thereby assaying the proportion of methylene-H_4_F generated via the long route through formate or from the direct route from formaldehyde. (B) Detection of serine by GC–MS. The small peak in total ion abundance detected by the MS denoted by the arrow represents serine. (C) Analysis of the mass fragments present in this peak revealed the presence of ions with M/z values of 156 and 228, which are diagnostic for ECF–TFAA derivatized serine.

A CD_3_OD label tracing strategy (Figure 2) was devised to directly determine what fraction of the methylene-H_4_F that entered the serine cycle was formed from the direct condensation of formaldehyde and H_4_F (direct route), versus the fraction formed through the alternative potential route involving oxidation of formaldehyde to formate by the H_4_MPT module, followed by assimilation through the H_4_F module (long route). The serine that is produced from methanol contains the carbon atom, and both hydrogens, from the methylene group of the methylene-H_4_F donor. Serine produced from CD_3_OD via the direct route contains two D, while that produced via the long route contains one D and in both cases these are relatively nonexchangeable C-D bonds. Therefore, at short labeling times (<1 min) the ratio of serine isotopomers with one or two D is an assay of the ratio of flux through the two routes.

In order for this label tracing method to be successful, the ratio of serine isotopomers containing one or two deuteriums from CD_3_OD must be determined. Initially, cultures were labeled with standard methanol (CH_3_OH), added to boiling ethanol after labeling, and the derivatized H_2_O-soluble small molecules were prepared and analyzed via gas chromatography–mass spectrometry (GC–MS). Consistent with a derivatized serine standard and previous work [22,23], a peak was observed at approximately 8.6 min that contained two major ions with M/z of 156 and 228 (Figure 2B and 2C). The proportion of (+1) and (+2) M/z ions detected were within 1.1% ± 1.7% and −0.7% ± 0.5% of the predicted distribution (Isoform 1.02, National Institute of Standards and Technology) of naturally occurring heavy isotopomers for these fragments, indicating the feasibility of this GC–MS method for detecting serine isotopomers.

### Deuterium Labeling Demonstrates Assimilation of C_1_ Units through Both Direct and Long Routes

Initially, the incorporation of deuteriums from CD_3_OD into serine was investigated with succinate-grown cell suspensions of wild-type M. extorquens AM1. Analysis of the derivatized H_2_O-soluble small molecule preparation from wild-type samples indicated a substantial increase in the proportion of fragments present as (+1) and (+2) isotopomers (>35% of total serine isotopomers). CD_3_OD labeling with a *glyA* mutant strain (CM239K.1), which lacks the initial serine-cycle enzyme, serine hydroxymethyltransferase, and was therefore completely unable to assimilate carbon from formaldehyde, produced no increase in (+1) or (+2) isotopomers (data not shown). Additionally, mutants defective for the proposed long route for methylene-H_4_F formation were tested for deuterium labeling. These included the *ftfL* (encodes formate-H_4_F ligase) mutant CM216K.1 [11], blocked for the H_4_F module, and the *dmrA* (encodes dihydromethanopterin reductase) mutant CM212K.1 [24], which has been shown to lack H_4_MPT [25,26]. Consistent with their proposed roles, the proportion of (+1) fragments dropped 8-fold for these mutants, compared to a modest 2-fold decrease in (+2) fragments. These data indicate that both the H_4_F and H_4_MPT modules affect labeling of serine and are required to generate the large increase in (+1) isotopomers seen with wild-type. These data also indicate that potential exchange reactions that could eliminate the deuteriums do not contribute measurably to the presence of (+1) ions. Collectively, these data indicate that the (+1) and (+2) serine mass fragments can serve as an accurate proxy for methylene-H_4_F generated through the long or direct routes. One caveat to this statement is that a portion of the NADPH involved in generating methylene H_4_MPT could be derived from the oxidation of methylene H_4_MPT to methenyl H_4_MPT and, therefore, could have become deuterium labeled. Based on the stoichiometry of the reactions and the known activity ratio of NADPH- versus NADH-producing enzymes for the methylene-H_4_MPT dehydrogenase reaction, we calculated that we at most overestimate the contribution of the direct pathway by 25% during growth on methanol, and by significantly smaller values at times with lower formaldehyde production. This prediction assumes an infinitely small intracellular concentration of NADPH, so depending on the actual pool of NADPH present, the error will be less. Therefore, our results are presented as maximum ratio changes.

When labeled with CD_3_OD, the succinate-grown wild-type cultures utilized to verify the GC–MS method produced a ratio of (+1) versus (+2) serine mass fragments of 8.0 ± 0.6. Thus, when succinate-grown cells are first exposed to methanol, the majority of methylene-H_4_F assimilated via the serine cycle is generated via the proposed long route. In contrast, CD_3_OD labeling of mid-exponential-phase methanol-grown cells indicated that the direct route dominated by up to 15-fold (measured ratio of [+1]/[+2] of 0.065 ± 0.006). Therefore, although both methylene-H_4_F production routes operated under both physiological conditions, a significant shift in the ratio of the two routes occurred, up to 100-fold.

### Relative Contributions of the Long and Direct Routes of Methylene-H_4_F Formation during Transitions to and from Methylotrophic Growth

**Figure 3:**
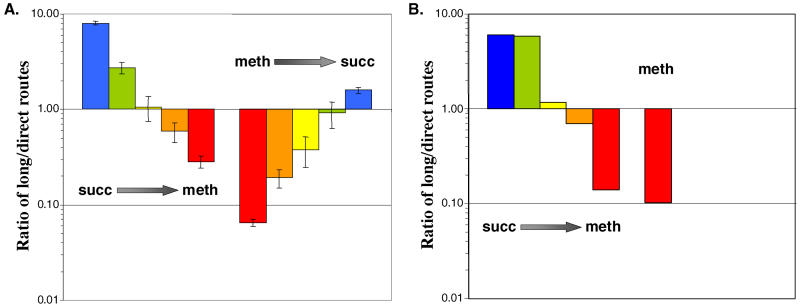
Change in Ratio of Flux through Long Versus Direct Methylene-H_4_F Formation Routes during Growth Transitions (A) Experimental data as determined by GC–MS analysis of serine isotopomers. The bars for each transition represent a time series from cells harvested 1 h prior to the transition, and four time points following the transition (succinate to methanol: 1, 5, 7.5, and 10 h; methanol to succinate: 1, 3, 5, and 7 h). (B) Predictions based on kinetic model simulations. The bars indicate the succinate to methanol transition (same time points as for the experimental data) and the methanol steady-state prediction.

In order to understand the dynamics of the contribution of the long and direct routes for directing C_1_ units into assimilatory metabolism during transitions to and from methylotrophic growth, metabolic shift experiments were performed. One hour after samples were removed from succinate- and methanol-grown cultures for the labeling experiments described above, the remaining portions of the two cultures were harvested, washed, and resuspended into medium containing the other substrate (methanol or succinate, respectively). At four intervals during the transition to each of the new growth substrates (Figure 3) samples were harvested and analyzed via CD_3_OD labeling to determine the ratio of flux capacity through the two methylene-H_4_F formation routes. The ratio of the contribution of the long route for methylene-H_4_F formation to the direct route varied in a continuous fashion during the transition from succinate to methanol, or from methanol to succinate (Figure 3A). The cultures were followed for 7 or 10 h after the shift—sufficient time to observe the majority of the transition.

### Dynamics of C_1_ Fluxes during Transitions between Succinate and Methanol by ^14^C Labeling

**Figure 4:**
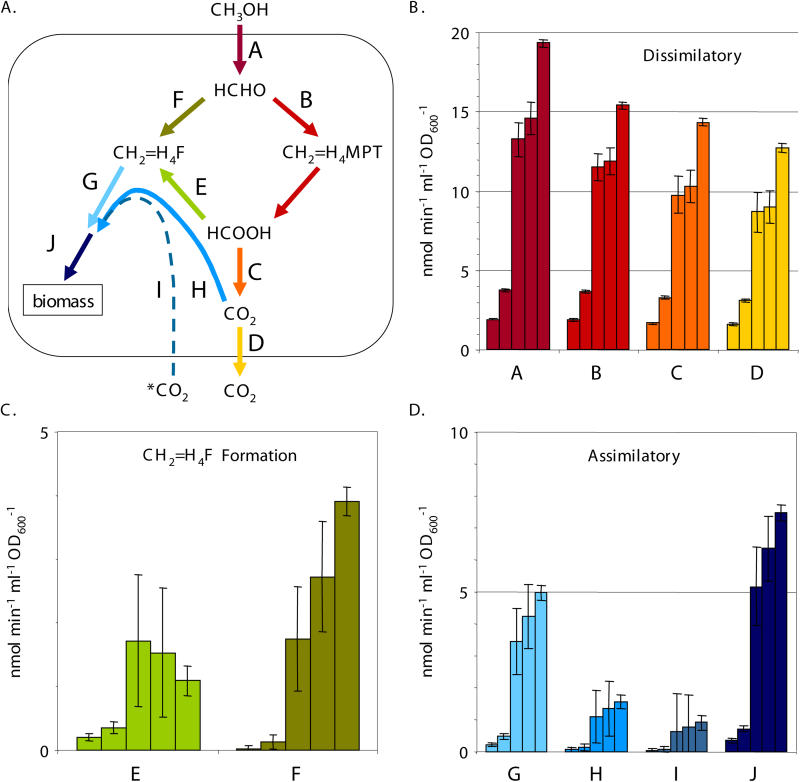
C_1_ Fluxes during Transition from Succinate to Methanol The fluxes determined are represented schematically (A). The other panels present flux for each branch, labeled A through J. The five bars for each flux represent a time series from cells harvested 1 h prior to the transition from succinate to methanol, and 1, 5, 7.5, and 10 h after the switch. Dissimilatory (B), methylene-H_4_F formation (C), and assimilatory (D) fluxes are presented separately with different scales for clarity. Flux F represents maximum fluxes.

**Figure 5:**
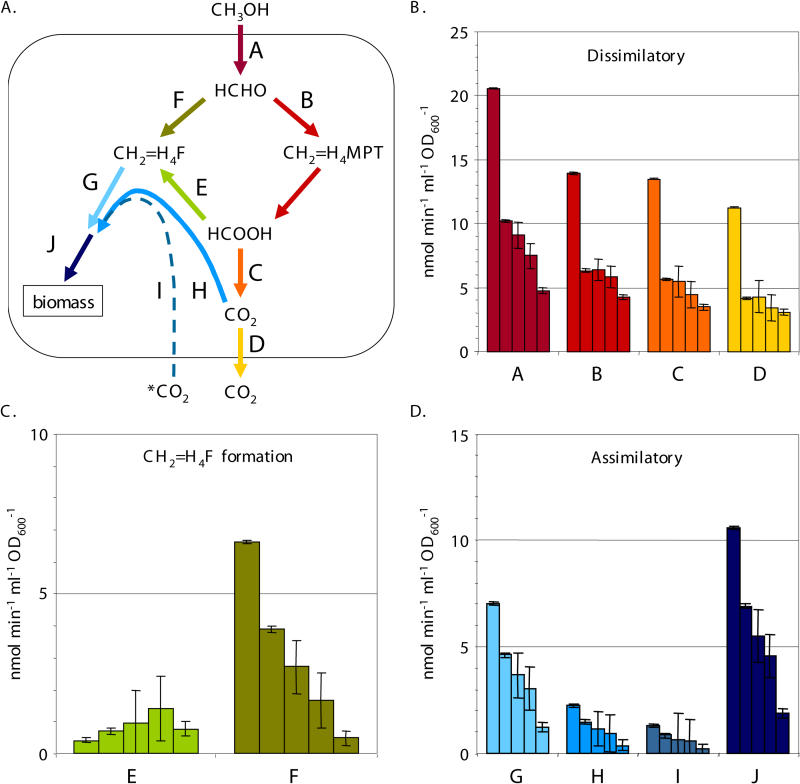
C_1_ Fluxes during Transition from Methanol to Succinate The fluxes determined are represented schematically (A). The other panels present flux for each branch, labeled A through J. The five bars for each flux represent a time series from cells harvested 1 h prior to the transition from methanol to succinate, and 1, 3, 5, and 7 h after the switch. Dissimilatory (B), methylene-H_4_F formation (C), and assimilatory (D) fluxes are presented separately with different scales for clarity. Flux F represents maximum fluxes.

The relative ratio of the routes provides only one of the parameters needed to understand the metabolic dynamics during this transition; the quantitative flux is also necessary. These values were obtained with ^14^C-labeling experiments. Concurrent with the CD_3_OD-labeling experiments described above, a portion of each sample was used to determine the rates of methanol oxidation, assimilation of C_1_ units, and CO_2_ production via ^14^C-CH_3_OH labeling [11]. Methanol oxidation was found to be 10-fold higher in methanol-grown cultures, and the percentage of carbon from methanol assimilated into biomass was 3-fold higher as compared to succinate-grown cultures (Table 1). The other values incorporated into the flux calculations are the stoichiometry of the serine cycle, in which two C_1_ units from methylene-H_4_F and one CO_2_ are incorporated for every C_3_ compound assimilated, and the proportion of external, unlabeled CO_2_ incorporated by the serine cycle [27]. The ten C_1_ fluxes (each branch arbitrarily labeled “A” through “J”) calculated using the concurrent CD_3_OD and ^14^C-methanol labeling methods are reported in Table 1 and shown in Figures 4 and 5.

**Table 1:**
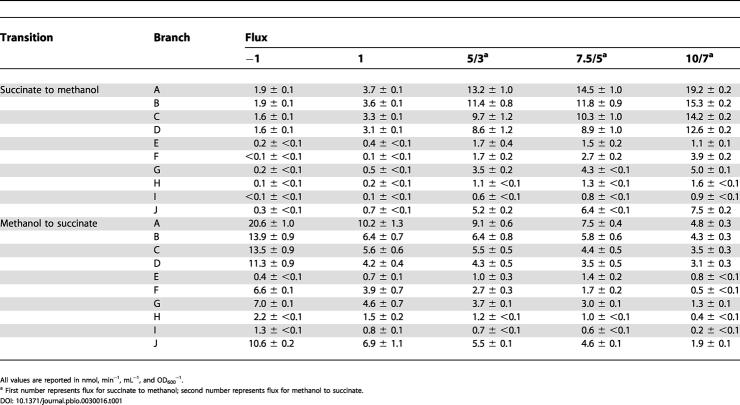
Calculated C_1_ Fluxes during Transitions between Succinate and Methanol at the Time (h) Relative to the Transition

All values are reported in nmol, min^−1^, mL^−1^, and OD_600_^−1^

^a^ First number represents flux for succinate to methanol; second number represents flux for methanol to succinate

A comparison of the values for succinate- versus methanol-grown cells shows that upon initial exposure of succinate-grown cells to methanol (Figure 4 and Table 1), the measurements suggest that most (at least 99%) of the formaldehyde was handled by the H_4_MPT module (flux B), and only a small amount flowed through the direct route (flux F). Of formate made from the H_4_MPT module (flux B), most (up to 88%) was converted to CO_2_ via formate oxidation (flux C), and a smaller amount (at least 12%) flowed through the H_4_F module and into assimilation (flux E), representing at least 90% of the assimilatory carbon. In contrast, for methanol-grown cells (Figure 5 and Table 1), less (only about 70%) of the formaldehyde generated from methanol flowed through the H_4_MPT module (flux B), with up to 30% handled by the direct route (flux F). Only a small portion of the assimilatory carbon (suggested to be about 6%) flowed through the H_4_F module (flux E), which represented about 3% of the formate generated via the H_4_MPT module. The remainder of the formate was oxidized to CO_2_ (flux C). These data indicate that, although the relative contribution of the long route to methylene-H_4_F formation decreased during the transition to growth on methanol (see Figure 3), the flux through the long route (flux E) increased significantly (see Figure 4). Flux through this route peaked 5 h after the transition to methanol, when it reached a value at least 8-fold higher than succinate-grown cells, and dropped somewhat afterward. The flux through the direct route (flux F) also increased to a maximum of up to 20% of the total formaldehyde flux at the final time point during the transition (see Figure 4). The fluxes for the transition from methanol to succinate represent the capacity for flux, as no methanol was present after the growth transitions. These changes, however, roughly mirrored the transition from succinate to methanol, but were not an exact reversal (see Figure 5). As noted for the deuterium-labeling experiments, the time periods followed in these experiments were sufficient to observe the majority of the transition.

### Dynamic Mathematical Model of Formaldehyde Partitioning

In order to assess whether the known kinetic constraints of the three modules of formaldehyde metabolism were sufficient to account for the experimentally determined flux dynamics, a mathematical model was generated. The model simulated partitioning of C_1_ units through the three formaldehyde modules during growth of cells in methanol, and for the transition of succinate-grown cells to methanol. The model consisted of eight ordinary differential equations, based on known kinetic mechanisms, to describe the dynamics of the H_4_F and H_4_MPT modules and the direct condensation reaction. Most binding constants, rate constants, and cofactor concentrations were obtained from the literature (Table 2). For the six cases in which literature values are not known, these were estimated as described in Materials and Methods. Additionally, a dynamic simulation of the succinate to methanol transition was performed. The methanol uptake rate was set to the experimentally measured value at each time point (flux A, Table 1) and interpolated linearly between time points to create a smooth gradient. Starting with the values obtained for succinate or methanol growth, the parameters were increased throughout the shift at a rate corresponding to the increase in methanol uptake.

**Table 2:**
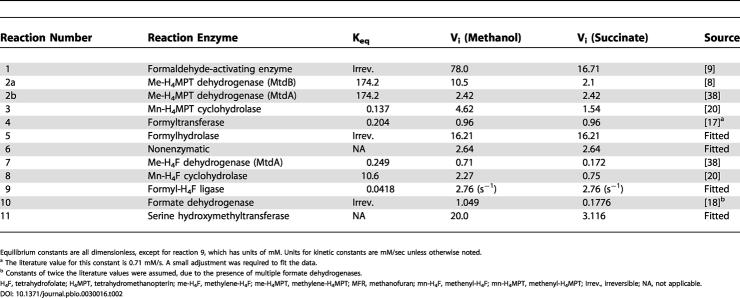
Equilibrium Constants and Forward Rate Constants (V_max_) for Each Reaction in the Model Simulation

Equilibrium constants are all dimensionless, except for reaction 9, which has units of mM. Units for kinetic constants are mM/sec unless otherwise noted

^a^ The literature value for this constant is 0.71 mM/s. A small adjustment was required to fit the data

^b^ Constants of twice the literature values were assumed, due to the presence of multiple formate dehydrogenases

H_4_F, tetrahydrofolate; H_4_MPT, tetrahydromethanopterin; me-H_4_F, methylene-H_4_F; me-H_4_MPT, methylene-H_4_MPT; MFR, methanofuran; mn-H_4_F, methenyl-H_4_F; mn-H_4_MPT, methenyl-H_4_MPT; Irrev., irreversible; NA, not applicable

Two key results are apparent from the comparison of the model's predictions (see Figure 3B) to the measured flux ratio of the two methylene-H_4_F production routes (see Figure 3A). First, the model did not constrain the direction of flux through the H_4_F module. Therefore the prediction that the H_4_F module functions in assimilation both during steady-state methanol growth and upon the first exposure of succinate-grown cells to methanol indicates that the kinetic parameters of the module components are sufficient to account for this phenomenon. Second, the correspondence between the predicted and experimentally determined dynamics of the switch in methylene-H_4_F production routes confirms that the dynamics of the system are also largely attributable to the systems' kinetic constraints. That the kinetics did not exactly mimic the measured values is presumably partly due to differences between the actual induction of enzyme activities versus the model's simplifying assumption that all values change in a manner directly proportional to changes in methanol uptake. However, the model does not suggest a significant effect of methylene H_4_MPT-derived NADPD in the deuterium-labeling studies.

### The H_4_F Module Could Not Be Eliminated during Growth on C_1_ Compounds

The combination of CD_3_OD and ^14^C-methanol label-tracing studies clearly demonstrate that the long route contributes methylene-H_4_F to the serine cycle and that the flux through the H_4_F module portion of the long route (flux E) increases significantly during the transition to growth on methanol. These results confirm the hypothesis of net reductive flux through this module [11,16,17]. However, this route contributes only 6% of the total methylene-H_4_F generated during growth on methanol. Therefore, it seemed possible that the H_4_F module might be required during transitions in and out of methylotrophy, but might not be required for continuous growth on methanol. Given the available genetic techniques, two strategies were employed in an attempt to obtain mutants in one of the key H_4_F module genes, formate-H_4_F ligase, during growth on C_1_ compounds. First, attempts were made to obtain null mutants via allelic exchange with cultures maintained on methanol or methylamine, but these efforts were unsuccessful. Second, cultures of the *ΔftfL::kan* mutant CM216K.1 [11] bearing the complementing plasmid pCM218 [11] were grown in medium containing methanol or methylamine without tetracycline for plasmid maintenance. No plasmid-free isolates were obtained for CM216K.1 with pCM218 during growth on methanol. However, they were obtained for wild-type with pCM218 on methanol, or CM216K.1 with pCM218 grown on succinate. Therefore, it appears that the H_4_F module plays an essential role in methylotrophy even after cells have already begun to grow on C_1_ compounds.

## Discussion

In the formaldehyde metabolism of M. extorquens AM1, three interconnected metabolic modules are present, involved in two roles: converting formaldehyde to the key assimilatory intermediate methylene H_4_F and net oxidation of formaldehyde to CO_2_. Understanding paradigms for differential roles of redundant modules is central to enabling broadscale metabolic reconstruction from genome sequences. In addition, methylotrophy represents an intriguing example of a metabolic mode in which growth depends on high flux of a toxic metabolite, with subsequent partitioning of that metabolite. Other such modes are known that produce toxic aldehydes, for instance, growth on ethanolamine [28] and other alcohols [29]. Numerous other toxic intermediates are known in bacteria, such as the production of hydroxylamine by ammonia-oxidizing bacteria [30] and mono-oxygenase-dependent production of epoxyalkanes during growth on aliphatic alkanes [31]. In addition, the liver can be exposed to toxic metabolites, for instance, the production of formate from acute methanol poisoning [32]. However, the metabolic mechanisms that allow the balancing of flux and toxicity in such situations are not well understood. Understanding paradigms for such metabolic responses is important for assessing and possibly ameliorating toxicity problems in a variety of systems, including bioremediation of toxic compounds, chemical production in bioprocesses, and detoxification in tissues and organs.

Through a combination of ^14^C and deuterium label-tracing strategies, we have defined flux through each metabolic module in methylotrophic metabolism in M. extorquens AM1 during transitions into and out of methylotrophy, in which the flux of formaldehyde into the system changed by a factor of 10. These methods had the dual advantages of possessing sufficient sensitivity to detect flux under all conditions tested, and being free from the requirement of steady-state growth conditions, which allowed the dynamics of growth transitions to be examined. Furthermore, this approach complements a recently developed ^13^C-labeling method that measures flux through the multicarbon branches of central metabolism [27], but is inherently silent to the C_1_ fluxes measured here. The approach described here allowed us to test and confirm the hypothesis that the role of the H_4_F module during growth on C_1_ compounds is to supply methylene-H_4_F from formate [11,16,17], although the fraction of total flux passing through this route is always small.

Given the small percentage of total flux into assimilation via the H_4_F module during growth on methanol, why is this module required under this condition? The results presented here suggest that this requirement is not alleviated even when cells begin to actively grow on methanol. It is possible that this module generates an inducing signal for the serine cycle and, therefore, is necessary to maintain assimilatory flux during growth on methanol. This hypothesis is consistent with the genetic circuit, as two of the genes encoding key enzymes of the H_4_F module *(mtdA* and *fch)* are in an operon with serine-cycle genes and are under the control of a single regulatory protein, QscR [33].

**Figure 6:**
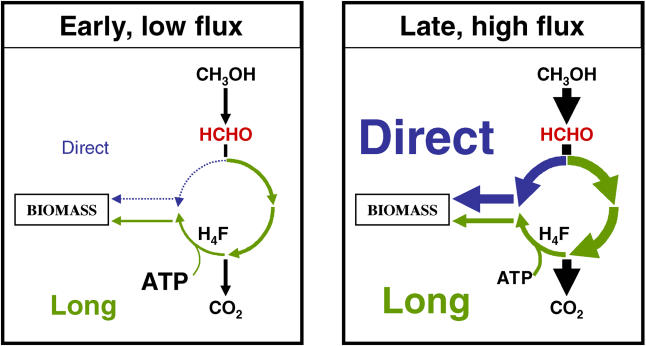
An Interconnected Metabolic Loop for Handling the Toxic Intermediate Formaldehyde A dynamic transition occurs from low to high formaldehyde flux, shifting the ratio of the direct versus long routes, and in the relative proportion of carbon oxidized to CO_2_ versus assimilated, creating a buffer system to accommodate large changes in formaldehyde flux.

Our results demonstrate a dramatic shift in flux through the primary methylotrophic modules during these transitions. It has long been known that all enzymes of methylotrophy increase 3–6 fold in activity after induction with methanol [14,16], predicting a sizable increase in total flux into the system. However, the flux measurements reported here show that a dynamic repartitioning occurs also. When M. extorquens AM1 encounters methanol, the methanol oxidation system is at low but significant activity [34]. Under these conditions, the flux of formaldehyde into the system is relatively low (Figure 6, left panel), and most of the formaldehyde is oxidized to CO_2_ via the H_4_MPT module and formate dehydrogenase, generating NAD(P)H. Only a trace amount is assimilated, almost all of that through the long route involving formate and H_4_F intermediates. As the flux of formaldehyde into the system increases, a greater percentage begins to flow through the direct route into assimilatory metabolism. A smooth transition occurs during the induction of the capacity in the system until approximately one-third of the total formaldehyde flows through this route, and assimilatory and dissimilatory metabolism are balanced for rapid growth on methanol (Figure 6, right panel). The metabolic elegance of this interconnected, dynamic metabolic loop creates an effective formaldehyde flux buffer for transitions, in which the cell has time to respond to the presence of a methylotrophic substrate, deriving benefit (energy) without risking buildup of a toxic intermediate. As the activity of the serine cycle begins to increase, more formaldehyde can be safely shunted to assimilatory metabolism via the direct, ATP-independent route, thereby ensuring the transition to growth on the C_1_ substrate without build up of formaldehyde.

What controls the rate of the nonenzymatic condensation of formaldehyde with H_4_F to form methylene-H_4_F, which was up to 150-fold greater during methanol growth than on succinate? The rate of this spontaneous reaction will be determined by the relative concentrations of reactants and products, with an equilibrium constant for this condensation of 3.2 × 10^−4^ [15]. Although this equilibrium constant favors the production of methylene-H_4_F, flux will only occur if either the concentrations of the reactants (formaldehyde and/or H_4_F) rise above the equilibrium concentration, or utilization of methylene-H_4_F is sufficient to keep the pool of this metabolite below the equilibrium concentration. At this time, it is not technically feasible to measure the intracellular concentrations of free formaldehyde or methylene-H_4_F. However, the most likely explanation for high flux through the nonenzymatic condensation of formaldehyde and H_4_F would be draw-off of the product (methylene-H_4_F) by the serine cycle. In order to test whether the known kinetic parameters explain the relative utilization of the two methylene-H_4_F production routes, a kinetic model was constructed and utilized to simulate formaldehyde partitioning during transitions to and from methylotrophic growth. The ability of the model to recapitulate the observed switch in route utilization (see Figure 3B) indicates that the architecture of the dynamic loop and the kinetic parameters of the responsible enzymes can predict operation of the H_4_F module in the assimilatory direction and are sufficient to account for partitioning of C_1_ units into assimilatory metabolism without accumulation of formaldehyde.

In summary, the dual-labeling approach described here for direct flux measurement during metabolic transitions has not only elucidated a key role for redundancy in the three metabolic modules responsible for formaldehyde assimilation and dissimilation, but has also revealed a new paradigm for accommodating high-flux toxic intermediates. It is likely that similar interconnected loop systems operate for other metabolites, toxic or not, and this example can now be used as a framework for predicting functions of other apparently redundant modules that may be involved in the handling of toxic metabolites.

## Materials and Methods

### 

#### Bacterial strains

Wild-type M. extorquens AM1 [35] and mutant strains were cultured at 30 °C in a minimal salts medium [36] containing 125 mM methanol or 15 mM succinate. A serine hydroxymethyltransferase mutant strain, CM239K.1 *(ΔglyA::kan)* was generated using the allelic exchange technique described previously [37].

#### CD_3_OD labeling and GC–MS

CD_3_OD (99.8%; Cambridge Isotope Laboratories, Andover, Massachusetts, United States) to a final concentration of 1 mM was added to washed cultures that had been resuspended to an OD_600_ = 1 in order to label cell metabolites with deuterium for analysis by GC–MS. After shaking for 20 s at room temperature the 2-ml suspension was added to three volumes of boiling 100% ethanol for instant lysis. Following centrifugation, the soluble fraction was dried, resuspended in distilled H_2_O, and centrifuged again to remove H_2_O-insoluble components. The resulting H_2_O-soluble small molecule fraction was then derivatized with ethyl chloroformate and trifluoroacetic acid as previously described [22,23]. All labeling experiments were performed three times.

#### GC–MS methods and data analysis

GC–MS experiments were performed using an Agilent 6890 gas chromatograph/Agilent 5973 quadrupole mass selective detector (electron impact ionization) operated at 70 eV equipped with an Agilent 7683 autosampler/injector (Hewlett-Packard, Palo Alto, California, United States). The MS was operated in selected ion monitoring mode to detect M/z = 156/157/158/228/229/230 from 7 min to the end of the method. The GC oven temperature started at an initial temperature of 60 °C, ramping at 20 °C min^−1^ to 130 °C, 4 °C min^−1^ to 155 °C, and then 120 °C min^−1^ to a final temperature of 300 °C that was held for 5 min. Flow through the column was held constant at 1 ml min^−1^. The injection volume was 1 μl and the machine was run in splitless mode. The temperature of the inlet was 230 °C, the interface temperature was 270 °C, and the quadrupole temperature was 150 °C. The column utilized was an HP-5MS (Hewlett-Packard).

GC–MS data were analyzed using Agilent Enhanced ChemStation G1701CA (Hewlett-Packard). The two mass clusters for serine, M/z = 156/157/158, and 228/229/230, represent fragments of ECF–TFAA derivatized serine (C_10_H_14_O_6_NF_3_) that have lost one or both of the carboxyl ethyl esters. The data were corrected for the natural abundance of heavy isotopes in the derivatized serine fragments, using proportions calculated with Isoform 1.02 (MS Search Program for Windows, National Institute of Standards and Technology, Gaithersburg, Maryland, United States). For each sample, the ratio of Δ + 1)/Δ + 2) was calculated for both mass clusters and averaged. The mean and standard error for these data were then calculated for the three replicates of each experiment.

#### Assimilation and CO_2_ production rates

The rate of ^14^C-CO_2_ production and assimilation of labeled carbon from ^14^C-methanol was determined concurrently with the CD_3_OD labeling described above using a modification of a previously described method [11]. A portion of the labeled cell suspensions was filtered (0.2 μM PVDF, Millipore, Billerica, Massachusetts, United States) to determine net assimilation. All measured and calculated fluxes were determined using the data from each of the three replicate experiments and then utilized to determine the mean and standard error for each flux.

#### Additional values incorporated into flux calculations

It has been determined previously that 63.3% of the total CO_2_ incorporated originates directly from CO_2_ produced from the oxidation of methanol [27]. This value cannot be determined under the nonsteady state conditions used in the experiments described here, so this value was incorporated directly into our calculations. The sensitivity of the calculated fluxes to a 2-fold increase or decrease in the determined ratio of 1.73:1.00 internal:external CO_2_ incorporated into the serine cycle was examined. Besides the direct effect on relative fluxes of internal and external CO_2_ into the serine cycle, the calculated incorporation of C_1_ units from methylene-H_4_F would vary no more than 7%, which would be balanced by a change in the dissimilatory flux through the H_4_MPT module and formate dehydrogenase of less than 6%. Therefore, deviations in the ratio of methanol-derived and external CO_2_ incorporation from the reported work [27] would not significantly alter the calculated fluxes.

#### Dynamic model

The dynamic model of the formaldehyde oxidation and assimilation modules consisted of eight ordinary differential equations, each describing the accumulation of a metabolite involved in the H_4_F and H_4_MPT modules. These equations were derived in a straightforward manner from the kinetic expressions given below. The production of formaldehyde from methanol was set to the measured rate of methanol uptake for each experiment. All enzymatic reactions were treated with either uni- or bimolecular reversible Michaelis–Menten kinetics, with the equilibrium constants taken from the literature [38]. In cases where K_eq_ > 200, the reverse reaction was ignored for simplicity. Finally, since the dynamics of serine and glycine were not included in this model, serine hydroxymethyltransferase was modeled as an irreversible unimolecular Michaelis–Menten reaction, with the effects of all metabolites other than methylene-H_4_F accounted for in an effective V_max_. The total internal concentrations of H_4_F and H_4_MPT derivatives were set equal to 0.15 and 0.4 mM, respectively [38]. Concentrations of other energy and redox cofactors (ATP, NADH, etc.) were assumed equal to those present in Escherichia coli [39]. The parameters used in the simulation are listed in Table 2. All K_m_s could be obtained from the literature (see Table 2), except for that of reaction 5. This K_m_ was set arbitrarily to 50 μM, which results in the reaction proceeding at half-maximal rate. Many of the values for V_max_ could be directly calculated from specific activities found in the literature, for both growth on methanol and succinate. To allow for experimental error in the measured rate constants, and to account for the fact that kinetics measured in vitro do not necessarily correlate exactly with what occurs inside the cell, these values were allowed to vary within 50% during the fitting procedure described below. For the remaining parameters, a numerical error minimization technique was used to find the set of parameters yielding model predictions with the best fit to the experimental flux distributions, when integrated to steady state. This was first done for methanol growth, then repeated for succinate growth. The rate constant for spontaneous formaldehyde condensation (k_6_) was forced to be the same on succinate as on methanol, since this is a fundamental chemical property that is not affected by gene induction. All reverse rate constants were calculated directly from the forward constants, binding constants, and Keq. The spontaneous condensation of formaldehyde with H_4_MPT was assumed to be negligible under physiological conditions compared to the formaldehyde activating enzyme reaction [9]. All simulations were performed in MATLAB 6.5 (MathWorks, Natick, Massachusetts, United States) using the ODE solving function “ode15s.” The error minimization was also done in MATLAB, using an evolutionary algorithm written previously [27].



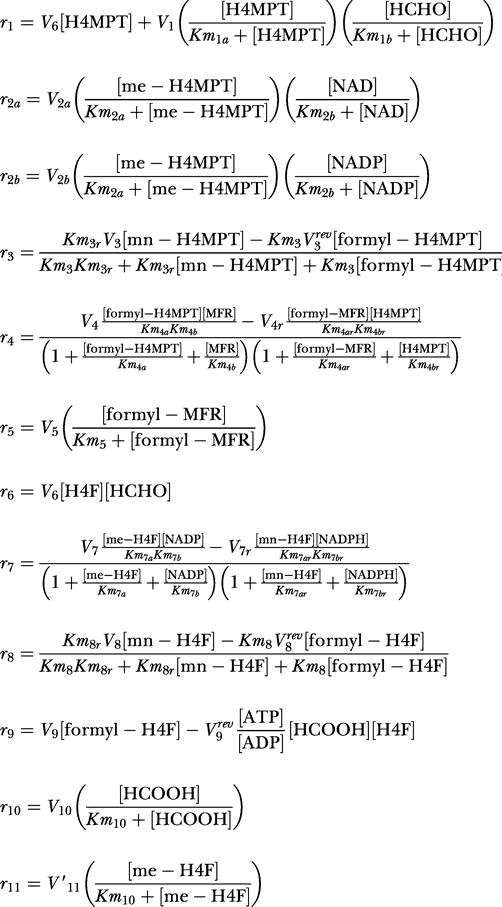



Abbreviations as in Table 2.

## Supporting Information

### Accession Numbers

The GenBank (http://www.ncbi.nlm.nih.gov/Genbank) accession numbers for genes discussed in this paper are *dmrA* (AY093431), *ftfL* (AY279316), and *glyA* (L33463).

## Abbreviations

GC–MS: gas chromatography–mass spectrometry
H_4_F: tetrahydrofolate
H_4_MPT: tetrahydromethanopterin
